# Origin and Evolution of Retinoid Isomerization Machinery in Vertebrate Visual Cycle: Hint from Jawless Vertebrates

**DOI:** 10.1371/journal.pone.0049975

**Published:** 2012-11-27

**Authors:** Eugenia Poliakov, Alexander N. Gubin, Olivia Stearn, Yan Li, Maria Mercedes Campos, Susan Gentleman, Igor B. Rogozin, T. Michael Redmond

**Affiliations:** 1 Laboratory of Retinal Cell & Molecular Biology, National Eye Institute, National Institutes of Health, Bethesda, Maryland, United States of America; 2 Biological Imaging Core, National Eye Institute, National Institutes of Health, Bethesda, Maryland, United States of America; 3 National Center for Biotechnology Information, National Library of Medicine, National Institutes of Health, Bethesda, Maryland, United States of America; University Zürich, Switzerland

## Abstract

In order to maintain visual sensitivity at all light levels, the vertebrate eye possesses a mechanism to regenerate the visual pigment chromophore *11-cis* retinal in the dark enzymatically, unlike in all other taxa, which rely on photoisomerization. This mechanism is termed the visual cycle and is localized to the retinal pigment epithelium (RPE), a support layer of the neural retina. Speculation has long revolved around whether more primitive chordates, such as tunicates and cephalochordates, anticipated this feature. The two key enzymes of the visual cycle are RPE65, the visual cycle *all-trans* retinyl ester isomerohydrolase, and lecithin:retinol acyltransferase (LRAT), which generates RPE65’s substrate. We hypothesized that the origin of the vertebrate visual cycle is directly connected to an ancestral carotenoid oxygenase acquiring a new retinyl ester isomerohydrolase function. Our phylogenetic analyses of the RPE65/BCMO and N1pC/P60 (LRAT) superfamilies show that neither RPE65 nor LRAT orthologs occur in tunicates (*Ciona*) or cephalochordates (*Branchiostoma*), but occur in *Petromyzon marinus* (Sea Lamprey), a jawless vertebrate. The closest homologs to RPE65 in *Ciona* and *Branchiostoma* lacked predicted functionally diverged residues found in all authentic RPE65s, but lamprey RPE65 contained all of them. We cloned RPE65 and LRATb cDNAs from lamprey RPE and demonstrated appropriate enzymatic activities. We show that *Ciona* ß-carotene monooxygenase a (BCMOa) (previously annotated as an RPE65) has carotenoid oxygenase cleavage activity but not RPE65 activity. We verified the presence of RPE65 in lamprey RPE by immunofluorescence microscopy, immunoblot and mass spectrometry. On the basis of these data we conclude that the crucial transition from the typical carotenoid double bond cleavage functionality (BCMO) to the isomerohydrolase functionality (RPE65), coupled with the origin of LRAT, occurred subsequent to divergence of the more primitive chordates (tunicates, etc.) in the last common ancestor of the jawless and jawed vertebrates.

## Introduction

Vertebrate vision depends on light-dependent isomerization of a chromophore (11-*cis* retinal) bound to the visual pigment opsin, a family of G-protein-coupled receptor (GPCR) proteins, triggering the phototransduction cascade, and resulting in neural signals being sent to the brain. These events are followed by the dissociation of the isomerized chromophore (all-*trans* retinal) from opsin. To regenerate the visual pigment chromophore, a process of continuous enzymatic isomerization, termed the visual cycle, is employed (for review see [1], [2]). In addition to the RPE-based “classical” visual cycle under consideration here, physiological evidence for a cone photoreceptor-specific visual cycle centered in the Müller glia cells has been accumulating (for review see [2]). However this cone-specific cycle has not been characterized at the molecular level, so its evolutionary origins cannot be addressed at the present time.

While the light-dependent reaction occurs in the photoreceptor cells, the enzymatic *trans*-to-*cis* re-isomerization occurs in the cells of the RPE, a monolayer epithelium adjacent to and partly enclosing the photoreceptor cells. In brief, the released all-*trans* retinal is reduced to all-*trans* retinol in the photoreceptor and then transported to the RPE where it is esterified by lecithin:retinol acyltransferase (LRAT) [3], to all-*trans* retinyl ester. The all-*trans* retinyl ester serves as substrate for the RPE65 isomerohydrolase [4], which converts it to 11-*cis* retinol. The latter is then oxidized by retinol dehydrogenase 5 (RDH5) in conjunction with CRALBP, an 11-*cis* retinoid-specific binding protein. The resultant 11-*cis* retinal is then returned to the photoreceptors to regenerate opsin. The proteins in the visual cycle of mammals and other higher vertebrates are mostly known and characterized. RPE65 acts as the key retinoid isomerohydrolase in the visual cycle [5], [6], [7]; mutations in this enzyme lead to retinal disease (Leber congenital amaurosis 2 (LCA2) and retinitis pigmentosa) resulting in blindness [8], [9]. LRAT is the obligatory source for all-*trans* retinyl esters, as its deletion in mouse [10] phenocopies the deletion of RPE65 [11].

Though it appears to be a conserved process in the vertebrate retina, the RPE-based visual cycle has not been established in lamprey, one of the most primitive extant vertebrates. Furthermore, the phylogenetic origin of the vertebrate visual cycle is still unclear. Recently, it was proposed that a prototype of the vertebrate visual cycle is operational in the tunicate *Ciona intestinalis* [12] when Tsuda and coworkers identified CRALBP, BCMO1 and opsin orthologs in *Ciona intestinalis* larva and a presumed RPE65 ortholog in adult animals [13]. Though these authors did not test for enzymatic activity of this presumed RPE65 ortholog, they later reported in a review article [14] that they could not detect such activity, though no data was presented. BCMO1 orthologs are also found in arthropods [15] and are essential for chromophore production [16], but this alone does not indicate a vertebrate visual cycle. While a CRALBP-like homolog is found in the Drosophila genome [17], its precise function and whether it can actually bind 11-cis retinal has not been determined. Mammalian RPE65 activity was demonstrated only after 12 years of thorough biochemical work and so the absence of activity for presumptive *Ciona* RPE65 in itself may not serve as evidence of different function. However, in neither case did they address whether LRAT was present or not. RPE65 is the only known member of the carotenoid oxygenase family to use retinyl ester instead of a carotenoid as substrate. Therefore, it is reasonable to hypothesize that an enzyme that could reliably provide this novel substrate for RPE65 would appear contemporaneously in evolution with an ancestral RPE65 to facilitate this new enzymatic function for a carotenoid oxygenase. To clarify these questions we performed phylogenetic analysis for both the RPE65 and the LRAT families. We found that a gene for an LRAT ortholog is not present in the curated genomes of either *Ciona intestinalis* or the cephalochordate *Branchiostoma floridae*. These results for non-vertebrate chordates are consistent with the *in silico* studies of Albalat [18]. However, we have extended these studies of Albalat [18] to provide experimental data for functions of these proteins. The first chordate LRAT orthologs we found were in the sea lamprey *Petromyzon marinus* (which has two copies of LRAT- LRATa and LRATb- as does the teleost *Danio)*. We confirmed our findings with determination of the enzymatic activity of the recombinant proteins and immunofluorescence studies of RPE65 in RPE, showing that functional lamprey LRATb and RPE65 are present in lamprey RPE. We also demonstrated that *Ciona* BCMOa (annotated as RPE65 in the *Ciona* draft genome) has carotenoid oxygenase cleavage activity, but no discernable RPE65 activity, rendering unlikely the premise that a vertebrate visual cycle arose before the last common ancestor of the jawless and jawed vertebrates.

## Results

### Phylogenetic Analysis of the RPE65/BCMO Superfamily

**Figure 1:**
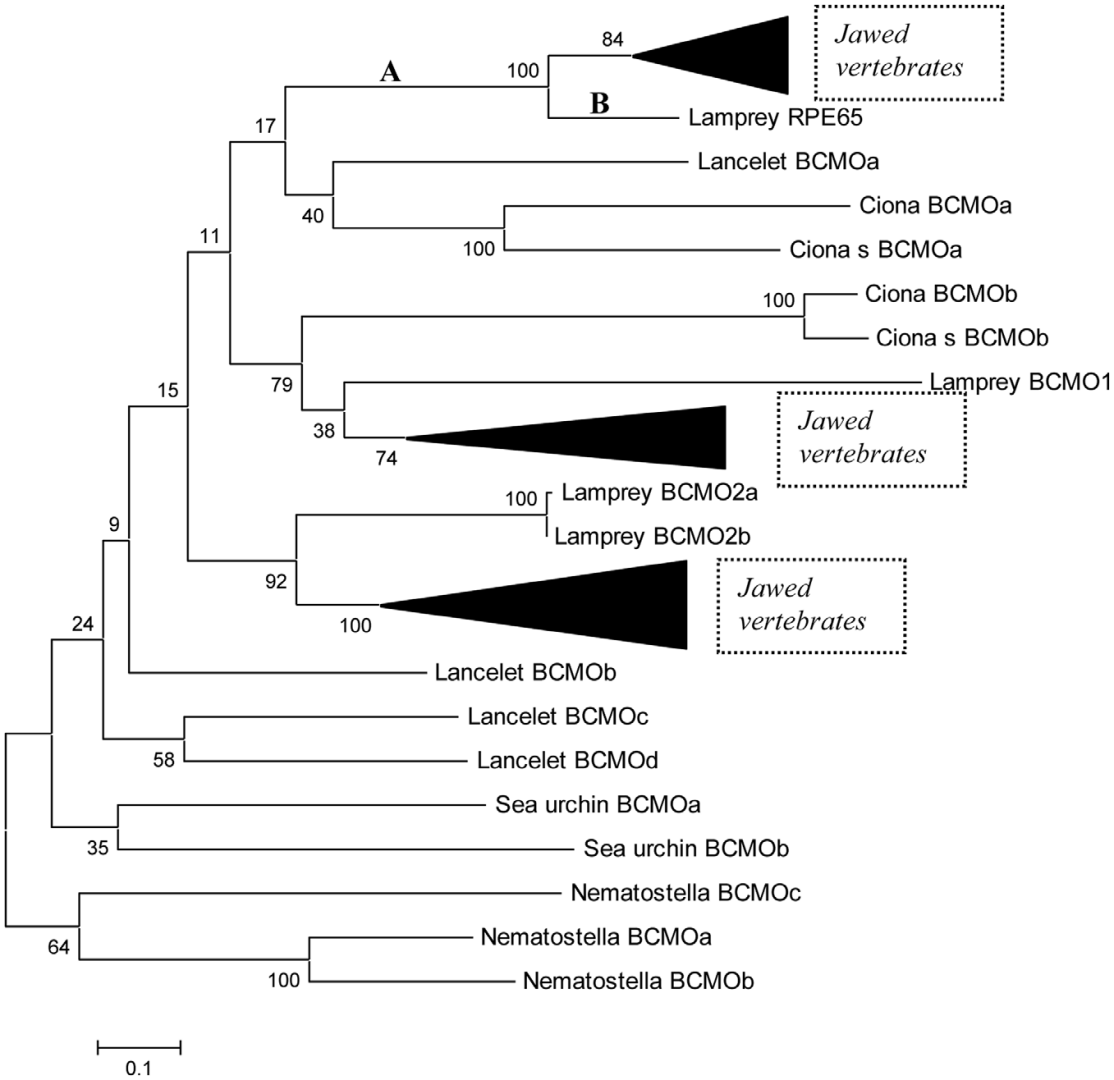
Maximum likelihood phylogenetic tree of the RPE65/BCMO superfamily (the WAG substitution model, the complete deletion option, the uniform rate of substitutions option as implemented in the MEGA5 program). The numbers for the interior branches refer to the bootstrap values with 1,000 pseudoreplicates. Ciona_s stands for *Ciona savignyi*.

**Figure 2:**
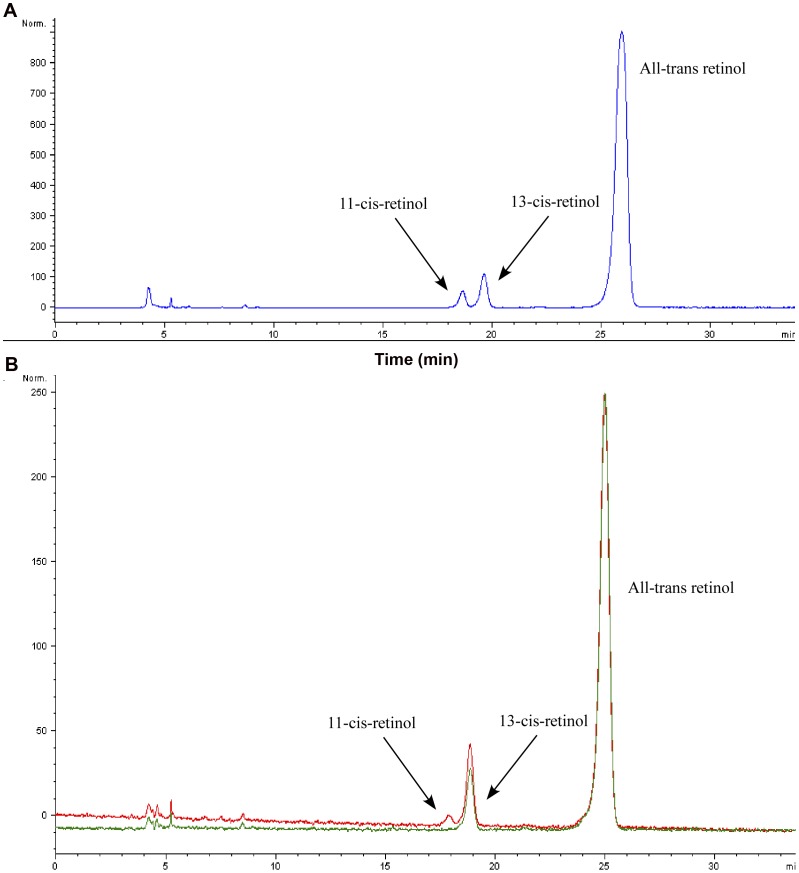
Production of 11-*cis* retinol by Lamprey RPE65 in HEK293F cells. A: Normal-phase HPLC of retinol isomers from saponified retinyl esters isolated from HEK293F cells expressing Lamprey RPE65 and bovine LRAT (*blue trace*). B: Normal-phase HPLC of retinol isomers from saponified retinyl esters isolated from HEK293F cells expressing Lamprey RPE65 with Lamprey LRAT (*red trace*) or only Lamprey LRAT (*green trace*).

A maximum likelihood (ML) phylogenetic tree of the RPE65/BCMO superfamily is shown in Figure 1. The topologies of ML, NJ (neighbor-joining), MP (maximum parsimony) and ME (minimum evolution) trees are slightly different- however these differences do not affect the results and conclusions of the phylogenetic analysis (Figure S1). The ML tree is rooted using sea anemone (*Nematostella vicentis*) BCMO sequences (Figure 1). The *Ciona* BCMOb sequence forms a well-supported clade with the vertebrate BCMO1 sequences (the bootstrap value is 79; Figure 1). The *Branchiostoma floridae* (Cephalochordata) BCMOa and the *Ciona intenstinalis/Ciona savignyi* BCMOa (Ci-RPE65) form a clade with the RPE65 family (Figure 1). However, the statistical support for this grouping is extremely low (the bootstrap value is 17). Furthermore, this clade is not observed in phylogenetic trees reconstructed using different methods (Figure S1). These results strongly suggest that this grouping is not reliable. This notion is consistent with the absence of this grouping in the RPE65/BCMO phylogenetic tree from the recent paper by Albalat [18]. As the vertebrate BCMO1 and BCMO2 families have numerous paralogs in fish genomes (Figure S1), species tree inferences and functional predictions are very complicated for these families. The phylogeny of the vertebrate RPE65 family follows the species tree with some deviations for fish-specific duplications (Figure S1), which suggests that all members of the family are true orthologs that perform the same function. This idea is further supported by experimental evidence for many vertebrate species, including the lamprey RPE65 (Figure 2a and Figure 2b).

The RPE65 family is separated from the rest of the tree by an extremely long branch (the branch A, Figure 1). This branch suggests that an ancestor of RPE65 experienced relatively fast evolution compared to other parts of the tree. The length of this branch is almost two times longer than the branch leading to the lamprey RPE65 sequence (the branch B, Figure 1). The branch A corresponds to approximately 50 million years, whereas the branch B corresponds to 500 million years [19], [20], [21], [22]. Thus the ancestor of RPE65 experienced ∼10 time faster evolutionary rate compared to the slow evolutionary rates of the RPE65 family (Figure 1). Such obvious acceleration of evolutionary rates is expected for proteins that are in the process of gaining a new function [23], [24], [25]. Based on this hypothesis, deuterostome carotenoid oxygenase proteins outside the RPE65 family do not have isomerohydrolase function and are likely to retain the original oxygenase activity since we did not find any other internal branches that experienced such dramatic acceleration of evolution (Figure 1). Thus, although the *Ciona intestinalis* BCMOa was initially annotated as RPE65 and was predicted by sequence alignment to be an isomerohydrolase [14], this is not supported by the branch length/time estimates presented in this study, or by our experimental evidence (see below).

Analysis of functionally important residues using DIVERGE2 (see Materials and Methods) suggested 7 residues that were substantially functionally diverged from the BCMO2 clade (divergence value >3 at positions L49, Q64, A92, K332, A415, L437, N451). We chose the BCMO2 clade for analysis because pairwise alignment of mouse BCMO2, BCMO1 and RPE65 proteins revealed more identities for the RPE65/BCMO2 and BCMO1/BCMO2 pairs than for the BCMO1/RPE65 pair (Table S1), and so we believe the RPE65 and BCMO1 clades diverged from the BCMO2 clade. Out of these 7 residues, four are the closest neighbors of residues that are critically important for the function of RPE65 [7], [26]. This result is not unexpected because it has been suggested by different authors that there is an evolutionary coupling between neighboring sites [27], [28], [29], [30], [31]. We estimated the significance of this observation using a list of 36 critical residues of RPE65 reported in the literature, taken from studies of pathogenic RPE65 single amino acid changes, and single amino acid changes significantly impairing RPE65 isomerohydrolase activity (more than 50%) in cell-based assay [7], [26]. The probability that 4 out of 7 residues are located in the region +/−1 of 36 crucial residues is 0.012, according to Fisher exact test. This result suggests that the majority of the predicted functionally diverged residues are responsible for the fine-tuning/adaptation of catalytic residues to the newly acquired function of an ancestral RPE65 enzyme. Analysis of the sequences annotated as the *Ciona* RPE65 homolog and the *Ciona* BCMO1 homolog (from genomes of *Ciona savignyi* and *Ciona intestinalis*) demonstrated the presence of only 1 out of 7 critical residues for RPE65 protein, similar to many deuterostome carotenoid oxygenases. The lamprey RPE65 sequence, on the other hand, contained all 7 conserved residues out of 7 predicted by DIVERGE2, while none of the carotenoid oxygenases of studied invertebrates or non-vertebrate chordates had more than 4 out of 7 critical residues. Albalat [18] chose 13 residues deemed functionally important based on the pathogenicity of mutations in these positions and conservation among RPE65 orthologs. He found that invertebrate and non-vertebrate chordate members of the RPE65/BCMO superfamily did not show conservation of these functionally important residues [18]. We found that Lamprey RPE65 had 11 out of these 13 residues with two changes in less conserved residues (N321E and T457H). Three of the 7 residues picked up by DIVERGE2 are the closest neighbors of functionally important residues picked by Abalat [18]. Taken together, these observations suggest that the Ciona homologs of carotenoid oxygenases have not diverged from pre-RPE65 members of the carotenoid oxygenase (RPE65/BCMO) superfamily, and thus Ciona does not possess its own RPE65.

### Phylogenetic Analysis of the LRAT Superfamily

**Figure 3:**
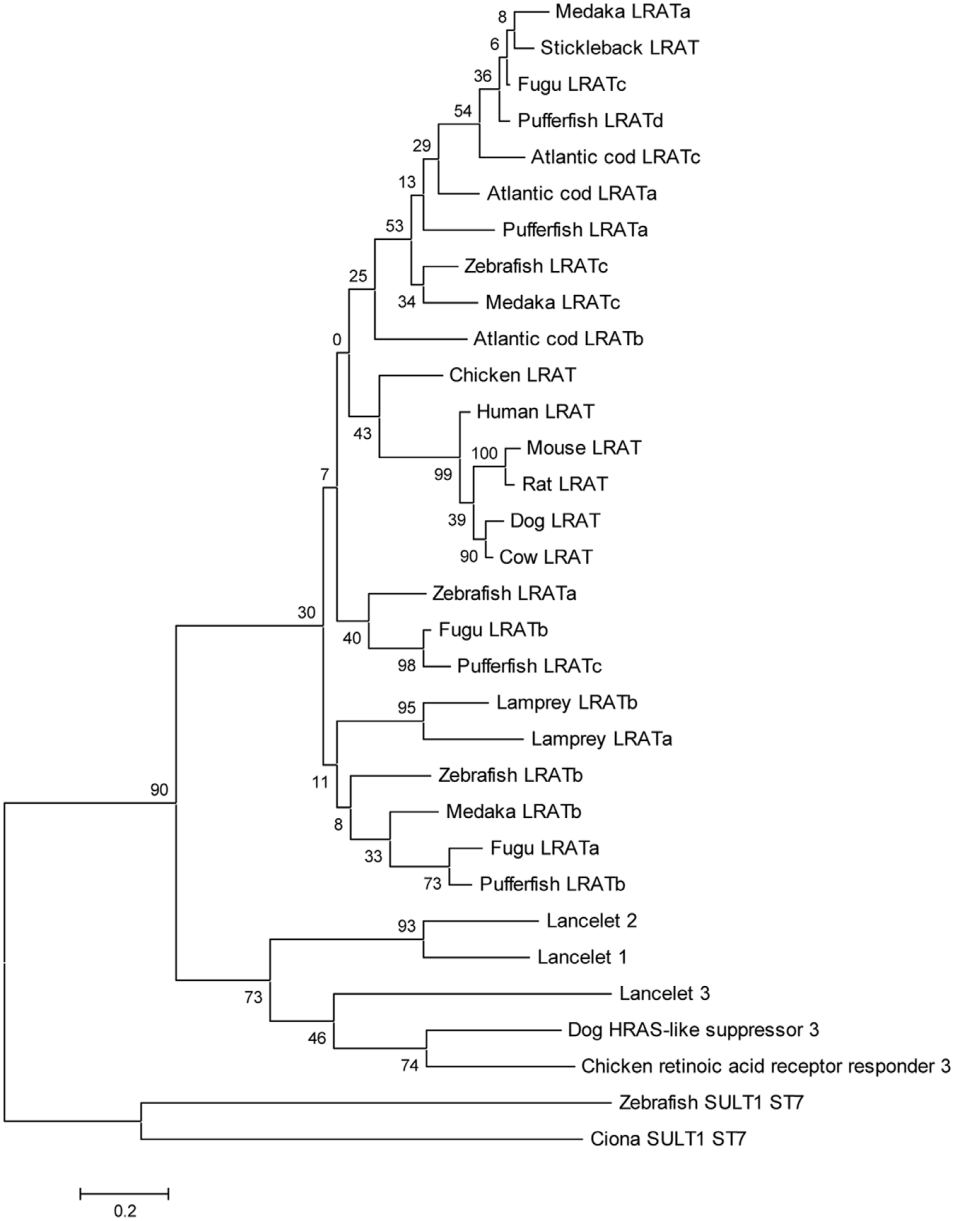
Maximum likelihood phylogenetic tree of the LRAT superfamily (the WAG substitution model, the complete deletion option, the uniform rate of substitutions option as implemented in the MEGA5 program). The numbers for the interior branches refer to the bootstrap values with 1,000 pseudoreplicates.

**Figure 4:**
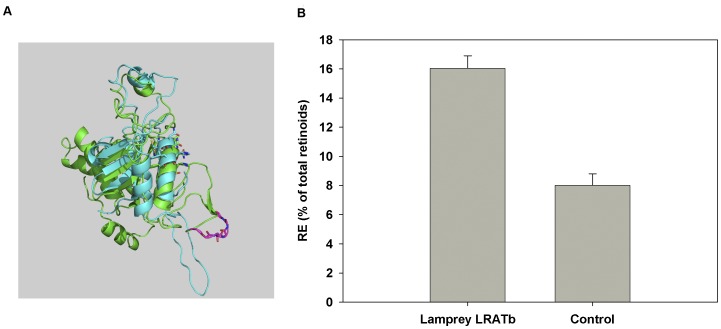
Functional LRAT is expressed in Lamprey RPE. A: Superposition of Lamprey LRATa and LRATb models on the H-REV107 crystal structure template (PyMol). *Blue*, Lamprey LRATa sequence. *Green*, Lamprey LRATb sequence. *Magenta*, 7-glycine motif on LRATb sequence. Side chains of a highly conserved LWNNCEHF motif with catalytic cysteine are shown in sticks, with *red,* oxygen; *blue* nitrogen and *yellow*, sulfur. B: Catalytic activity of Lamprey LRATb in HEK293F cells in the presence of specific DGAT1 inhibitor (A922500, 50 µM). Activity is expressed as ratio of retinyl esters to total retinoids (retinols and retinyl esters in %).

A maximum likelihood (ML) phylogenetic tree of the N1pC/P60/LRAT superfamily [32] is shown in the Figure 3. A few homologous sequences (SULT1-ST7, retinoic acid responder 3 and HRAS-like suppressor 3) were included in the LRAT alignment. We did not find any likely orthologs of LRAT in the *Ciona* genome; the closest LRAT homolog was the SULT1-ST7 protein, belonging to a different clade of N1pC/P60/LRAT superfamily (Figure 3). NJ, MP and ME trees are included in Figure S2. The tree topologies of ML, NJ and ME trees are not substantially different. The ML tree is rooted using the *Ciona intestinalis* and zebrafish SULT1-ST7 sequences (Figure 3). Vertebrate LRAT sequences form a clade (the bootstrap value is 30, a weak support; Figure 3) that is separated from the rest of the tree by a relatively long branch. However, a minimum evolution tree (Figure S2) suggested a much stronger support for the LRAT clade (highly significant support, 98%). This difference is likely to be due to relatively long branches leading to some LRAT homologs (e.g. SULT1-ST7). Such long branches are known to be a general problem for phylogenetic analysis, the so-called long branch attraction [33], [34], [35]. Although there are some deviations from the species tree (for example, lamprey LRATa/b forms a clade with four fish LRAT sequences, a poorly supported clade, Figure 3), the phylogeny of the vertebrate LRAT family in general follows the species tree (Figure 3). It is important to note that in all additional phylogenetic trees (ML, ME, NJ, and MP, Figure S2) lamprey LRAT sequences form an outgroup clade with respect to the other vertebrate LRAT sequences, and this grouping is consistent with the species tree. This suggests that many (if not all) members of the family are true orthologs performing the same (or very similar) function(s). This conclusion is further supported by experimental evidence for many vertebrate species including the lamprey LRAT (Figure 4b) and by phylogenetic trees reconstructed for the N1pC/P60/LRAT superfamily by Albalat [18].

### Catalytic Activity of *Ciona* BCMOa (*Ci-*RPE65), *Ciona* BCMOb (*Ci-*BCMO1) and Lamprey BCMO2a and BCMO2b

To determine *Ciona* BCMOa (previously annotated as ciRPE65 [13]) activity, we first cloned it into the pVITRO2/CRALBP vector [7] and transiently co-transfected with the pVITRO3/bovine LRAT/bovine RDH5 construct into HEK293-F cells. No isomerohydrolase activity was detected (data not shown). However, when the *Ciona* BCMOa (ciRPE65) open reading frame was cloned into the bacterial pBadTOPO vector and expressed in lycopene- or β-carotene-accumulating *E. coli,* the amount of lycopene (data not shown) or β-carotene in induced cells decreased significantly compared to uninduced cells (Figure S3 A and B). Quantification of β-carotene or lycopene in induced cell culture transformed with *Ciona* BCMOb (ci-BCO) also demonstrated significant carotenoid cleavage activity (Figure S3 B). No retinal was detected in extracts of ciBCMOa or ciBCMOb pointing to a BCMO2-like type of eccentric carotenoid cleavage (data not shown). This finding indicates that Ci-RPE65 possesses carotenoid oxygenase cleavage activity. Lamprey BCMO2a (Genbank/EBI accession number JX115002) and BCMO2b (Genbank/EBI accession number JX115003), having only 6 amino acid differences between them, were also subcloned into pBADtopo vector and transformed into lycopene or β-carotene accumulating *E.coli.* Expression of proteins in soluble form was confirmed by immunoblot analysis with the monoclonal His-tag antibody (Roche) (data not shown). No carotenoid oxygenase cleavage activity was detected for either (Figure S3 B).

### Catalytic Activity of Lamprey RPE65 and Lamprey LRATb in the HEK293-F Based Minimal Visual Cycle System

**Figure 5:**
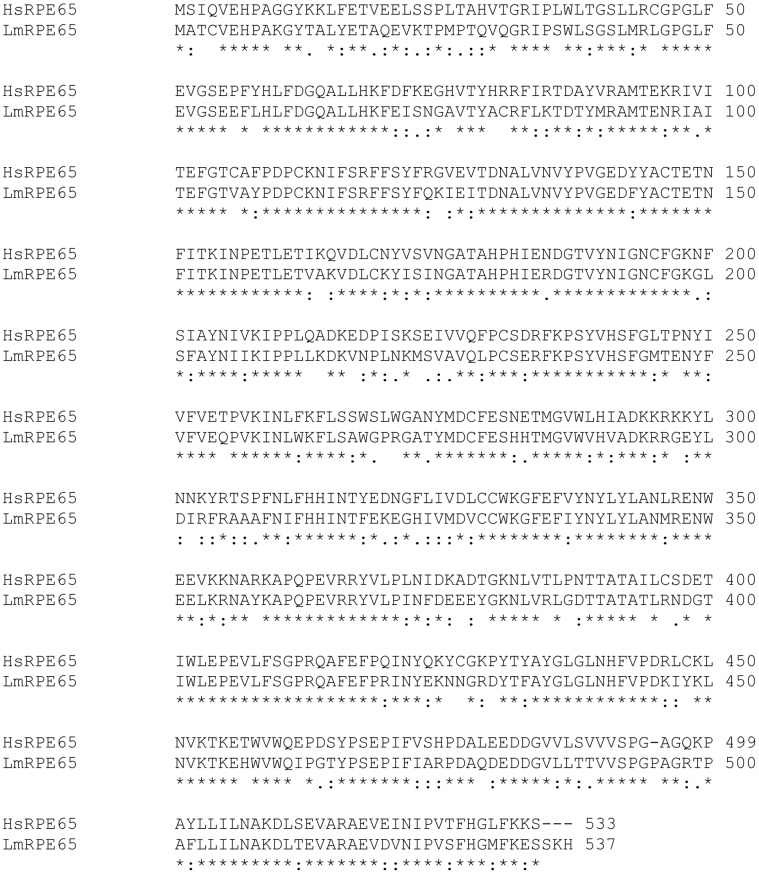
Alignment of Human and Lamprey RPE65. CLUSTAL W (1.83) alignment of Human RPE65 and Lamprey RPE65. GenBank/EBI accession numbers are as follows: human RPE65, NP_000320, lamprey RPE65 JX115001. *Red*, conserved residues around catalytic cysteine.

**Figure 6:**
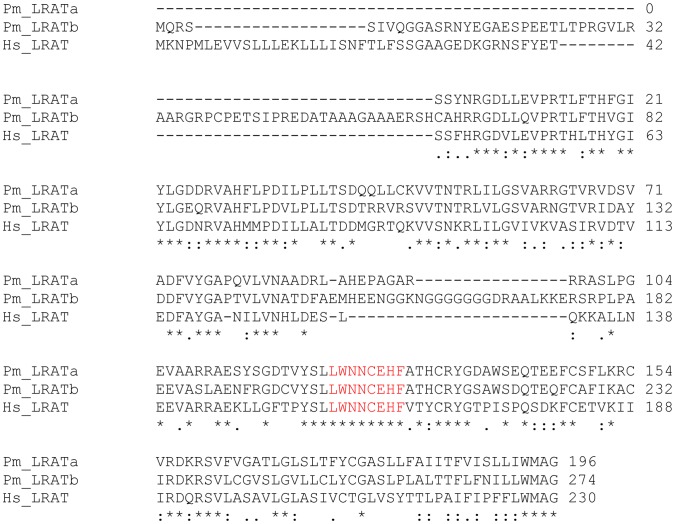
Alignment of Human and Lamprey LRAT sequences. CLUSTAL W (1.83) alignment of Human LRAT**,** Lamprey LRATa (partial sequence from contig9067, *Petromyzon marinus* Genome draft assembly WUSTL v.3.0 (March 2007) and Lamprey LRATb. GenBank/EBI accession numbers are as follows: human LRAT, AAH31053**,** lamprey LRATb, JX115000. Asterisks, identity; double dots, strong physico-chemical similiarity; single dots, weak physico-chemical similiarity.

To study the biochemical functions of lamprey RPE65 and lamprey LRAT we extracted total RNA from frozen RPE of adult female lamprey (*Petromyzon marinus*). The lamprey genome contains one copy of RPE65 and two copies of LRAT. We amplified and cloned RPE65 (Genbank/EBI accession number JX115001) and LRATb (Genbank/EBI accession number JX115000) from RPE total RNA (we could not amplify LRATa from RPE; Figure 5 and 6). Activity of lamprey RPE65 was assayed in the HEK293-F cell based minimal visual cycle assay as described previously [7]. Cells transfected with lamprey RPE65 and the bovine LRAT when treated with all-*trans* retinol produced 11-*cis* retinol (Figure 2a). Cells transfected with lamprey RPE65 and lamprey LRATb were also able to produce 11-*cis* retinol, however cells transfected only with lamprey LRATb did not produce 11-*cis* retinol (Figure 2b).

The *Petromyzon* LRATb protein contains a very interesting polyglycine tract (aa 160–166∶7 Gly in a row) in its primary sequence. As the polyGly tract raised a question about the functionality of this protein, we modeled lamprey LRATa and LRATb on the H-REV107 crystal N-terminal structure (2KYT). The quality of the models obtained is comparable: QMEAN4 score is 0.277 for LRATa and 0.242 for LRATb. Thus it seems that the polyglycine tract does not interfere with the catalytic active site (Figure 4a).

Diacylglycerol acyltransferase 1 (DGAT1) is an alternate retinyl ester synthetase capable of esterifying retinol in a variety of cells [36], [37]. We have confirmed the presence of mRNA for endogenous DGAT1 acyl transferase in HEK293F cells (data not shown). To distinguish between lamprey LRATb retinol esterification activity and the possibility of DGAT1 activity contributing to retinol esterification in the HEK293 assay, we performed our assay in the presence of 50 µM A922500, a DGAT1 specific inhibitor (Figure 4b).

### Immunohistochemistry and MALDI-TOF Analysis of RPE65 Protein in Sea Lamprey RPE

**Figure 7:**
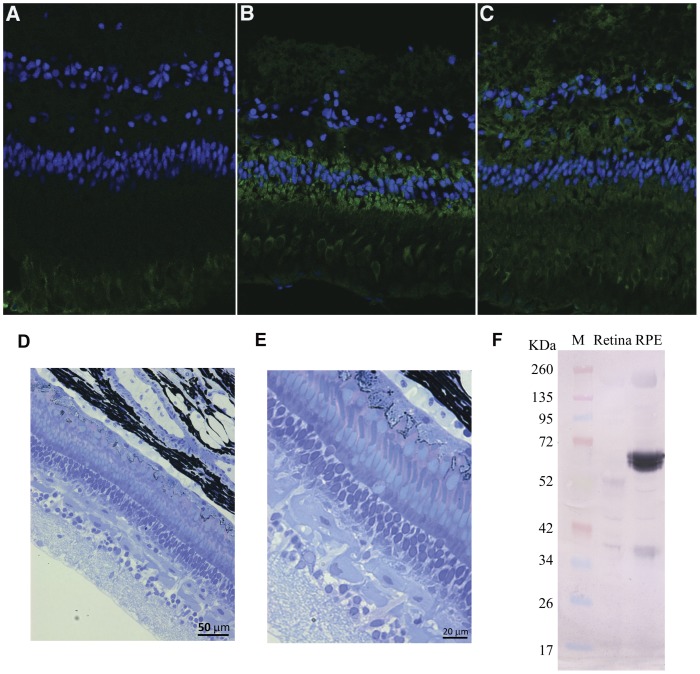
Localization of RPE65 in the Lamprey RPE/retina. Cy3 (green) staining of frozen sections of fixed lamprey retina/RPE with rabbit polyclonal antibodies to A: RPE65; B: arrestin; C: blue cone opsin SWS2 [38], [39]. Nuclear DAPI staining in blue. Lamprey retina histology D: 20X magnification; E: 40X magnification of semithin sections stained with toluidine blue. F: Immunoblot of Lamprey retina and RPE extracts. Retina and RPE were prepared as described in Methods. Lanes from left to right: *lane 1*, marker Spectra Multicolored Broad Range Protein ladder (Fermentas), *lane 2*, retina extract, *lane 3*, RPE extract.

Frozen sections of fixed lamprey retina/RPE were incubated with polyclonal rabbit antibodies to RPE65, visual arrestin, and blue cone opsin (SWS2) [38], [39] and visualized with Cy3 conjugated secondary anti-rabbit IgG (green signal) (Figure 7a, b, c respectively). RPE65 was clearly immunolocalized in Lamprey RPE (Figure 7a). Lamprey retina histology was visualized with toluidine blue stain (Figure 7d, e). Western blots probed with polyclonal rabbit antibody to RPE65 (“PETLET” epitope; [40]) revealed a prominent band at approximately 61 kDa in RPE (Figure 7f). We next sought to confirm the identity of this band as RPE65 by MALDI-TOF mass spectrometry. The Sea Lamprey genome is not annotated in the GenBank database and therefore standard mass fingerprinting is not possible. However, using MS-Digest (http://prospector2.ucsf.edu/prospector/cgi-bin/msform.cgi?form=msdigest) we predicted a peptide profile for Sea Lamprey RPE65 (537 aa, protein Mw 61.4 kDa) that would be generated by trypsin proteolysis. We matched 16 peptides in the trypsinized RPE65 immunoreactive band to our RPE65 predicted peptide set, with less than 0.1 Da difference and sequence coverage of 29% (Table 1). This confirmed the identity of the immunoreactive band as lamprey RPE65.

**Table 1:** MALDI-TOF Lamprey RPE65 peptide mass fingerprinting.

Centroid mass	Theoretical mass	Difference (Da)	Relative intensity	Peptide
796.4328	796.4312	0.0016	5.31	360–366
877.4239	877.4203	0.0036	45.69	414–420Gln-pyrroGlu
894.4414	894.4468	−0.0054	66.58	414–420
933.493	933.4941	−0.0011	18.19	264–271
1114.549	1114.567	−0.0182	4.93	24–33
1128.484	1128.518	−0.0336	3.45	acetyl1–10
1130.58	1130.562	0.0174	10.29	24–33met ox
1246.659	1246.661	−0.0025	15.72	34–44
1262.673	1262.656	0.0164	11.6	34–44metox
1319.642	1319.645	−0.0025	2.54	223–234
1419.693	1419.622	0.0716	7.49	321–332
1678.865	1678.866	−0.0009	35.54	171–185
1715.835	1715.816	0.0187	12.66	368–381
1759.891	1759.892	−1E-04	4.76	306–320
1871.939	1871.917	0.0211	20.87	367–381
1929.955	1929.971	−0.0159	16.97	397–413
1956.944	1956.949	−0.0048	6.29	430–446

Theoretical monoisotopic masses for Lamprey RPE65 trypsin-generated peptides were determined by MS-Digest.

### Possible Photosiomerases in Lamprey Genome

In order to address the question of RPE65 independent visual pigment regeneration we checked for the presence/absence of RGR/peropsin genes in the lamprey using BLASTP searches. We first ran control experiments using known *Ciona* RGR and *Branchiostoma* (lancelet) peropsin [41]. Symmetrical best BLASTP hits (protein × in 1^st^ species finds protein Y in the 2^nd^ species as the top BLASTP hit and protein Y in 2^nd^ species finds protein × in the 1^st^ species as the top BLASTP hit) are frequently used as a definition of orthologous proteins [23], [24], [25]. Both proteins found human RGR/peropsin proteins as symmetrical best hits (Table S2). For the lamprey proteins we used a more relaxed definition of orthology: we analyzed the three best BLASTP hits of human RGR/peropsin in the lamprey proteome instead of one best hit. We ran BLASTP searches of these proteins against the NR protein database (www.ncbi.nlm.nih.gov) and analyzed the best hits in vertebrates (Table S2). All six hits were not RGR/peropsin proteins (Table S2). This result strongly suggests that there are no obvious orthologs of RGR/peropsin genes in the lamprey genome. It is likely they have been lost as there are orthologs of these genes in Ciona and lancelet (Table S2). There is a possibility that these genes remain unsequenced or unassembled. However, the chances of this are not great taking into account that these are long multi-exon genes.

## Discussion

Key to the unique structure of the vertebrate eye is the inverted neural retina, and its adjacent support cell layer, the RPE. While vertebrates continued to use the ancient photosensitive GPCR opsin family as its visual pigments, a radical departure was made to regenerate the 11-*cis* retinal chromophore by a “dark” enzymatic process, rather than by an evolutionarily more commonly used photoisomerization process. We suggest here that this enzymatic process, or visual cycle, arose uniquely by evolution or co-option of proteins at the same time as the vertebrate eye evolved, perhaps as an adaptation to facilitate higher visual performance in dim light or in situations of sudden change from dark to light conditions compared to its ancestral precursors. We now show that jawless vertebrates (lamprey), in common with jawed vertebrates, have functional RPE65 and LRAT in their RPE. The previous finding of two functional visual opsins that are regenerated with 11-*cis* retinal also supports the presence of a fully functional vertebrate visual cycle in lamprey [42]. It seems plausible that fully functional RPE65 and LRAT were already present in the last common ancestor of jawed and jawless vertebrates, but that such organisms, and their immediate precursors, have been lost to evolutionary history.

The tunicates, including *Ciona*, have recently displaced the cephalochordates as the closest known extant relatives of vertebrates. However, it is well accepted that the tunicate lineage is diverged from the line leading from the ancestral chordates to the last common ancestor of the jawless and jawed vertebrates. This has not discouraged efforts to discern ancestral aspects of features common to the vertebrate lineage. The vertebrate eye with its visual cycle is one such feature. Our experimental evidence supports Albalat’s [18] view that the cephalochordate (*Branchiostoma*) and tunicate (*Ciona*) proteins related to vertebrate visual cycle components are probably not involved in chromophore regeneration. This, together with the phylogenetic analysis of RPE65 and the absence of LRAT makes us conclude that *Ciona intestinalis* does not have a visual cycle comparable to vertebrates. As we do not have any indications that a prototype of the vertebrate visual cycle was secondarily lost in *Ciona intestinalis*, it likely never evolved. In fact, Nakashima *et al.* [43] conclude that 11-*cis* retinal in the *Ciona* larval ocellus is supplied from Ci-opsin3, a photoisomerase opsin, suggesting reliance on the more primitive pathway. Larval *Ciona* also expresses Ci-opsin1, a ciliary-type opsin as its visual pigment [13], [44]. Furthermore, the expression of Ciona BCMOa/Ci-RPE65 occurs in the sessile adult stage where non-visual roles, such as phototropism, siphon contraction and gamete release [13] have been proposed for photoreception, and not in the free-swimming larval stage. Ci-opsin3 is also expressed in the adult neural complex along with Ci-CRALBP and the BCMOa/Ci-RPE65. If BCMOa/Ci-RPE65 is not capable of isomerizing retinol, as we have found, then this role can be accomplished by the photoisomerase RGR opsin homolog Ci-opsin3 in both developmental stages. (Conversely, we could not detect any photosiomerases (peropsin or RGR opsin homologs) in the lamprey genome that could potentially accomplish RPE65-independent visual chromophore regeneration.) The presence of a CRALBP-like protein in *Ciona* (Ci-CRALBP) suggests that trapping and transport of 11-*cis* retinal derived from Ci-opsin3 could occur in both the larva and adult stages. Though Ci-CRALBP clusters with vertebrate CRALBP (unlike *Branchiostoma* CRALBP-like homolog [13], which clusters with α-tocopherol transfer protein (aTTP; data not shown), another member of the CRAL-TRIO family [45]), it has not yet been shown experimentally to actually bind 11-*cis* retinal. The absence of a robust LRAT ortholog (other than a SULT1-ST7-like homolog) in *Ciona* further weakens the case for a patent visual cycle in ascidians. Thus, we conclude that *Ciona* does not possess a coherent retinoid metabolic pathway that is comparable with the vertebrate visual cycle. To reiterate, our main rationale for this view is the absence of functional RPE65 and LRAT orthologs.

Given the evident absence of a vertebrate-like visual cycle in the pre-vertebrate chordates, it was important to establish its earliest origins in the most primitive vertebrates. Various components of the vertebrate retina phototransduction system have been found in the lamprey including opsins [39], [42], photoreceptor-specific transducins [46], and photoreceptor-specific cyclic nucleotide phosphodiesterase 6 (PDE6) [47]. However, until now, components of the vertebrate visual cycle had not been identified in lamprey. We find that lamprey RPE65 is remarkably similar to mammalian RPE65 (72% identity/92% similarity), indicating that most, if not all, of the transition to a functional isomerohydrolase from a BCMO2-like ancestor had already occurred by the last common ancestor of jawless and jawed vertebrates. This was borne out by the phylogenetic tree and DIVERGE2 analyses, and the strong immunoreactivity of lamprey RPE65 to the anti-human RPE65 antibody. This suggests that once a functional RPE65 was achieved, further evolutionary modification was minimal. Co-expression of LRAT with RPE65 is crucial for a working visual cycle [7], [10]. Therefore it was important that we also established the presence of LRAT in lamprey, its earliest occurrence in evolution. Since a clear precursor to the vertebrate visual cycle does not appear to exist in the more primitive chordates, it must have evolved after these taxa diverged from the line leading to vertebrates, but by the last common ancestor of the jawless cyclostomes (lampreys and hagfishes) and the jawed vertebrates. While many morphologists formerly held the view that hagfishes are more primitive than lampreys, various molecular phylogenetics datasets (microRNA families, ribosomal DNA, mitochondrial DNA, etc.) strengthen the viewpoint that the cyclostomes are monophyletic [48], [49]. This means that the most primitive vertebrate eyes known are found, collectively, in lampreys and hagfishes, with the proviso that the degenerate eyes of hagfish, among other features, are a secondary acquisition. In fact, a gradient of degeneracy is seen among eyes of hagfishes [50]. Alternatively, hagfish may be an arrested or neotenous form of lamprey development and, accordingly, their eyes may correspond to an early stage of vertebrate eye development [51]. Thus the vertebrate eye is a so-called primitive character of vertebrates and seems to have appeared “from nowhere”, along with all the other vertebrate primitive characters. In reality, intermediates stages including the last common ancestor of the jawed and jawless vertebrates and its immediate precursors would appear to have been lost since their putative origin in the Cambrian explosion over 500 million years ago and are not known, so far, in the fossil record [52]. This means that the question of the origin of the vertebrate eye and its visual cycle, among a host of other vertebrate characters, is even more difficult to resolve. However, we can conclude that the crucial transition from typical carotenoid double bond cleavage functionality to the isomerohydrolase functionality, coupled with the origin of LRAT, occurred subsequent to divergence of the more primitive chordates (tunicates, etc.) from the line leading to vertebrates. Both carotenoid oxygenases and N1pC/P60/LRATs comprise multigene superfamilies with several paralogous genes per genome, thus it is likely that ancestors of LRAT and RPE65 emerged as a result of gene duplications, traditionally considered to be a major evolutionary source of new protein functions in eukaryotes [53], [54], [55], [56]. Studies of paralogous genes at the genome scale showed a substantial acceleration of evolution in all copies of recently diverged paralogs compared to orthologs with the same level of synonymous sequence divergence [23], [24]. This acceleration may be explained by positive selection or by a relaxation of purifying selection or by a combination of the two [23], [24]. Although the most likely outcome of such accelerated evolution is for one of the paralogs to fix a nonsense mutation and become a pseudogene, fixation of mutations (during a relatively short period of evolution) that lead to a new function also occurs [23], [24], [25]. Interestingly, a theoretical evaluation of the time required to evolve a camera type eye from a simple eyespot (assuming availability of photoreceptor cells, their necessary biochemical underpinnings (visual cycle, phototransduction cascade, etc.), and neural pathways to a brain) has suggested a pessimistic estimate of but a few hundred thousand years [57]. These and other considerations suggest that the first functional RPE65 and LRAT appeared in the last common ancestor of jawed and jawless vertebrates as the result of relatively fast evolution of duplicated copies of ancestral genes followed by acquisition of new functions.

## Materials and Methods

### Ethics Statement

Sea Lamprey tissues for this study were collected under an Animal Study Protocol approved by the National Eye Institute (NIH) Animal Care and Use Committee.

### Datasets and Phylogenetic Analysis and Modeling

Protein sequences were downloaded from the NCBI and ENSEMBL web sites. Similarity searches were performed using the non-redundant protein sequence database at the NCBI and the gapped BLAST program. Multiple protein sequence alignments were constructed using the Muscle program and then adjusted by hand (details available upon request from Eugenia Poliakov, Poliakove@nei.nih.gov). Phylogenetic trees based on multiple alignments of protein sequences were constructed using the maximum-likelihood, neighbor-joining, minimum-economy and maximum-parsimony methods as implemented in MEGA [58], FASTTREE [59] and PAUP* programs [60], [61]. A statistical method for estimating type-II (cluster-specific) functional divergence of protein sequences implemented in the DIVERGE2 program [62] was used for analysis of functionally important residues (vertebrate RPE65 and BCMO2 clades were used for analysis). The lamprey LRAT structure was modeled on the Swiss-Model server using the H-REV-107 crystal structure (PDB ID: 2KYT) as the template [63]–[64], [65]. DIVERGE2 was designed to detect functional divergence between member genes of a protein family based on (site-specific) shifted evolutionary rates after gene speciation or duplication. Posterior analysis results in a site-specific profile for predicting amino acid residues that are responsible for functional divergence. Moreover, when the 3D protein structure is available, these predicted sites are mapped to a 3D structure viewer to explore its structure basis [62].

### Cloning of ciBCMOa (ciRPE65) for Expression in *E.coli*

The ciBCMOa open-reading frame was obtained from a synthetic pUC57/ciBCMOa construct (Genscript, Piscataway, NJ) by amplification using Takara Taq polymerase. The resultant PCR product was directly cloned into the pBadTOPO vector (Invitrogen). The sequencing of the resulting pBadTOPO construct confirmed that the inserted DNA fragment was ciBCMOa in the correct orientation and position.

### Cloning of Lamprey LRAT and RPE65 for Expression in HEK293F Cells

RPE was carved out from frozen lamprey heads. Total RNA from lamprey RPE was purified using TRIzol® reagent (Invitrogen) according to the manufacturer’s instructions. Briefly, 2 RPEs were homogenized in 1 ml of TRIzol and incubated at room temperature for 5 minutes. 0.2 ml of chloroform was added and the tube was shaken for 15 seconds following by 3 minutes incubation at room temperature. The sample was centrifuged at 12, 000×g for 15 minutes at 4°C. The upper aqueous phase was removed and placed into a new tube. 0.5 ml of 100% isopropanol was added to the aqueous phase and mixed. After 10 minutes incubation at room temperature the sample was centrifuged at 12, 000×g for 10 minutes. The supernatant was removed from the tube and the RNA pellet was washed with 1 ml of 75% ethanol. The sample was vortexed briefly and centrifuged at 7500×g for 5 minutes at 4°C. The wash was discarded and the RNA was air dried for 5 minutes. The RNA pellet was resuspended in 20 µl RNase-free water.

SMARTer™ RACE cDNA Amplification kit (Clontech) was used to clone lamprey RPE65 and LRAT following manufacturer’s instructions. Phusion Flash II DNA Polymerase (Finnzymes) was used for PCR amplification.

For RPE65 cloning, 1 µg of total RNA from RPE was reverse transcribed in 10 µl reaction by SMARTScribe™ Reverse Transcriptase with 5′-RACE CDS Primer A 5′–(T)25VN–3′ and SMARTer II A Oligonucleotide 5′–AAGCAGTGGTATCAACGCAGAGTACXXXXX–3′ at 42°C for 90 min. This first-strand reaction product was diluted with Tricine-EDTA buffer to 100 µl. The recommended program for touchdown PCR was used with Phusion™ Flash High-Fidelity PCR Master Mix (Finnzymes) with Universal Primer A Mix (UPM) Long (0.4 µM), Short (2 µM) and lamprey_RPE65 5′-RACE primer (5′-GACAAGGATGAGGGAGGCCCAACTCGTAG-3′) that was designed based on partial genomic DNA sequence from contig39407, *Petromyzon marinus* Genome draft assembly WUSTL v.3.0 (March 2007). A ∼1.7 kb DNA single band was cloned into pCR-Blunt vector (Invitrogen) according to the manufacturer’s instructions, transformed into TOP10 competent cells, and grown on agar plates supplemented with kanamycin. Sequencing of plasmid DNA from several clones containing the 1.7 kb DNA fragment confirmed lamprey RPE65 identity. Lamprey RPE65 ORF was PCR amplified with Phusion Flash II DNA Polymerase and following primers: LamRPE65F:

5′-AAAGCAACCGGTGATATCATGGCTACTTGTGTGGAGCACCCTG-3′ and LamRPE65R: 5′-ACGCGTGGATCCGATATCCTAGTGCTTCGAGCTCTCCTTGAAC-3′. A 1.5 kb PCR product was cloned into the EcoRV site of pVITRO2-hygro-mcs expression vector (Invivogen) with cloned bovine CRALBP using the In-Fusion PCR cloning system (Clontech) following manufacturer’s instructions, transformed into TOP10 competent cells, and grown on agar plates supplemented with hygromycin. The resulting construct was confirmed by sequencing.

For lamprey LRAT cloning, total RNA from lamprey RPE (10 µg) was treated with Terminator™ 5′-Phosphate-Dependent Exonuclease (EPICENTRE Biotechnologies) to degrade ribosomal RNA. The remaining mRNA was concentrated using RNA Clean and Concentrator™-5 (Zymo Research) and reverse transcribed by SMARTScribe™ Reverse Transcriptase as previously described for RPE65. The first-strand reaction product was diluted with Tricine-EDTA buffer to 100 µl. The same touchdown PCR program as for RPE65 amplification was used in a reaction mix containing Phusion™ Flash High-Fidelity PCR Master Mix (Finnzymes) with Universal Primer A Mix (UPM), long (0.4 µM), short (2 µM) and lamprey LRAT 5′-RACE primer 5′-AGCGTTGGTGAGGAGGTGTCCTGGT-3′ (designed from lamprey partial genomic DNA sequence from contig9067, *Petromyzon marinus* Genome draft assembly WUSTL v.3.0 (March 2007)). A single 1.1 kb DNA band was obtained. This PCR product was cloned into pCR-Blunt vector (Invitrogen) according to the manufacturer’s instructions, transformed into TOP10 competent cells, and grown on agar plates supplemented with kanamycin. The cloned 1.1 kb DNA fragment was sequenced and confirmed to contain lamprey LRAT. The LRAT ORF was PCR amplified with Phusion Flash II DNA Polymerase and the following primers: LamLRAT2_InF_For:

5′-CACCCGGGCACCATGCAAAGGAGCAGCATTGTGCAGGGC-3′ and LamLRAT2_InFRev: 5′-TGCTCCTAGGCGTACTTACCCAGCCATCCACAGGAGGAT-3′, producing an 852 bp PCR product. This DNA fragment was inserted into NcoI and BsiWI sites of pVITRO3-mcs expression vector (Invivogen) using the In-Fusion PCR cloning system (Clontech) following manufacturer’s instructions, transformed into TOP10 competent cells, and grown on agar plate supplemented with hygromycin. The sequencing of the resulting pVITRO3_LRAT construct confirmed that the inserted DNA fragment was LRAT in the correct orientation and position.

Lamprey BCMO was cloned from the same RNA sample and using the same conditions as for LRAT cloning, with Universal Primer A Mix (UPM) and lamBCMO 5′-RACE primer 5′- GGTCGTCGTTATTAGACGACGTTGGGAGCG -3′ (designed from partial genomic DNA sequence from contig6156, *Petromyzon marinus* Genome draft assembly WUSTL v.3.0 (March 2007)). A single 2.0 kb DNA band was obtained. This PCR product was cloned into pCR-Blunt vector (Invitrogen) according to the manufacturer’s instructions, transformed into TOP10 competent cells, and grown on agar plate supplemented with kanamycin. Two clones were sequence confirmed to contain 2 variants of lamprey BCMO. The Taq-polymerase (Takara) amplified PCR products were directly cloned into pBadTopo vector. The sequencing of the resulting BadTOPO constructs confirmed that the inserted DNA fragments were lamprey BCMOa and lamprey BCMOb in correct orientation and position.

### BCMO1 Enzymatic Activity (Carotenoid Cleavage Activity)

BCMO1 enzymatic activity was assayed as described previously [66]. In short, the pBAD/ciRPE65 construct, under control of the arabinose promoter, was transformed in a lycopene-producing or a carotene-producing strain of *E.coli* (50 mL cell culture). Each culture was split in half after reaching OD_600_ = 0.6 and one-half was induced with 0.002% arabinose. After incubation for 18 hours, cells were harvested in 50 mL plastic tubes and color of the cell pellet was compared with uninduced controls. ß-carotene was extracted and quantified using reverse phase HPLC as described previously [66]. Lycopene was quantified using reverse phase HPLC as described previously [67].

### Transient Transfection and Cell Culture

Cell culture methods and transient transfection protocols have been previously published [7]. In a typical experiment, 3×10^7^ 293-F (Invitrogen, Carlsbad, CA) cells were transfected with 30 µg of pVitro2 plasmid (containing RPE65 (lamprey, chicken or dog) and CRALBP open reading frames (ORFs)) and 30 µg of pVitro3 (Invivogen) plasmid (containing lecithin-retinol acyl transferase (bovine or lamprey LRAT) in the presence of 60 µl of 293fectin transfection reagent (Invitrogen), all in a total volume of 30 ml. 24 hours after transfection, all-*trans* retinol was added to a final concentration of 2.5 µM and the cells were cultured for a further 5 hours and then harvested for analysis.

Presence of DGAT1 in HEK293F cells was confirmed by Bioanalyzer (Agilent) sizing experiment on DNA 1000 chip with Fwd HS DGAT1 S 5′-CCAGAACTCCATGAAGCCC-3′ and Rev HS DGAT1 AS 5′-TGTAGAAGTGTCTGATGCACC-3′ primers). cDNA was made from 4 µg total RNA by Retroscript kit (Ambion). Total RNA was prepared with RNAEasy mini kit (Qiagen). DGAT1 specific inhibitor A922500 (Tocris) was added to cells to a final concentration 25–50 µM from a 10 mM stock in DMSO, together with all-*trans* retinol.

### Retinoid Extractions and HPLC

Culture fractions of 20 ml volumes of transfected 293-F cells were centrifuged and cells were harvested and retinoids extracted and saponified as previously described [7]. Isomeric retinols were separated on a 3 micron YMC silica normal phase column (4.6×150 mm) and in-line 5 micron particle Lichrospher (Alltech, Deerfield, IL) normal phase column (4.6×250 mm) and analysed on an isocratic HPLC system equipped with a diode-array UV-visible detector (Agilent 1100/1200 series, Agilent Technologies, New Castle, DE), following Landers and Olson [68] as modified by us [7].

### Histology and Immunofluorescence Microscopy of Lamprey RPE65

Lamprey eyes were enucleated, pierced at the limbus, and fixed in freshly prepared 4% paraformaldehyde in phosphate buffered saline (PBS, pH 7.3). For plastic embedding, eyes were fixed overnight at 4°C before embedding. Semi-thin sections were cut and stained with toluidine blue. For immunofluorescence microscopy, eyes were fixed for 2 hours, then washed 3 times for 10 minutes each in ICC buffer (0.5% BSA, 0.2% Tween 20, and 0.05% sodium azide in PBS, pH 7.3) and cryo-protected in sequential 5%, 10%, 15% and 20% sucrose in ICC buffer containing 0.05% sodium azide, 1 hour each, or until the eyes sank. Following this they were embedded in Optimal Cutting Temperature (OCT) compound (Tissue-Tek® 4583 SAKURA) 2 parts and 20% sucrose 1 part, frozen in cold acetone and stored at -80°C until cutting. For immunofluorescence, 10 µm thick sections were cut and kept frozen until use. Prior to use, the sections were dried *in vacuo* for 30 minutes, washed 3 times with 1X PBS and blocked for 1 hour in 5% normal goat serum in ICC buffer. Following this, sections were incubated overnight in primary antibody solution (RPE65, blue cone opsin, red cone opsin or arrestin; all 1∶200 in ICC). The sections were then washed 3 times with 1X PBS, and incubated in Alexa 488-conjugated goat anti-rabbit IgG (1∶300) plus DAPI (1∶1000) in ICC. After final series of washes the slides were coverslipped and sealed with Fluoro-Gel with EMS.

### Immunoblot and MALDI-TOF Analysis of Lamprey RPE65

Lamprey RPE and retina were taken from adult female animal and frozen immediately. RPE (retina) was homogenized in a glass homogenizer on ice in Cytobuster buffer (EMD-Novagen) (1 mL) with complete protease inhibitors (1 mini tablet per 10 mL of buffer, Roche). Samples were prepared for SDS-PAGE. Denatured samples were separated on 10% BisTris NuPage (Life Technologies) gels and either stained by Coomassie Blue G and excised from gel for in-gel trypsin digestion or electrotransferred to nitrocellulose membranes. In-gel digestion was done as recommended by the Applied Biosystems Voyager manual with several changes. Trypsinization was done for 15 min at 50 W in a focused microwave device (CEM). Extracted peptides were purified on Vivapure C18 micro columns (Sartorius Stedim Biotech) and analyzed by a matrix-assisted laser desorption ionization-time of flight (MALDI-TOF) method (Voyager-DE STR, Applied Biosystems). Blots were probed with antibodies by standard procedures and developed in color substrate BCIP/NBT Phosphatase substrate (KPL). Primary antibodies used were: rabbit anti-bovine RPE65 antibody (1∶4000) [11]; Secondary antibody used was alkaline phosphatase-conjugated goat anti-rabbit IgG (1∶10,000; EMD-Novagen).

## Supporting Information

Figure S1 **Phylogenetic trees of the BCMO/RPE65 superfamily.** This shows tree topologies reconstructed using different phylogenetic methods. The numbers for the interior branches refer to the bootstrap values with 1,000 pseudoreplicates. Ciona_s stands for *Ciona savignyi*. **A**: ML, maximum likelihood phylogenetic tree, the WAG substitution model (this is the full version of Figure 1 without collapsing of the RPE65, BCMO1 and BCMO2 clades); **B**: ML, maximum likelihood phylogenetic tree, the JTT substitution model; **C**: NJ, neighbor-joining, the JTT substitution model; **D**: ME, minimum evolution, the JTT substitution model; **E**: MP, maximum parsimony.
(TIF)

Figure S2 **Phylogenetic trees of the LRAT superfamily.** This shows tree topologies reconstructed using different phylogenetic methods. The numbers for the interior branches refer to the bootstrap values with 1,000 pseudoreplicates. **A:** ML, maximum likelihood phylogenetic tree, the JTT substitution model; **B:** ME, minimum evolution, the JTT substitution model; **C:** NJ, neighbor-joining, the JTT substitution model; **D:** MP, maximum parsimony.
(TIF)

Figure S3 **Color shift due to the cleavage of β-carotene in** ***E. coli.*** **A.** This illustrates the color shift of the β-carotene-producing and -accumulating *E. coli* strain from orange to light yellow caused by the cleavage by BCMOa (Ci-RPE65) enzymatic activity of β-carotene to form apocarotenoids. While the induction of BCMOa (Ci-RPE65) expression partially bleaches the induced *E. coli* β-carotene strain within 18 hours (right tube), the uninduced CiRPE65 transformed culture remains orange (left tube). **B.** Quantification of β-carotene degradation in β-carotene-accumulating *E.coli*. Separate replicate cultures of cells were transformed with Lamprey BCMO2a, Lamprey BCMO2b, Ciona BCMOa (ci-RPE65), or Ciona BCMOb (ci-BCO), grown to OD_600_ = 0.6, split in half, then one-half was induced with 0.02% arabinose and each half allowed to grow overnight. Then cells were collected and β-carotene and its degradation products were extracted and analysed by reverse phase HPLC as described in Materials and Methods.
(TIF)

Table S1 **Number of identities in triple and pairwise alignments of mouse BCMO2, BCMO1 and RPE65.** T-coffee alignment of the three proteins, taking gaps into consideration.
(DOC)

Table S2 **Best BLASTP hits of human RGR and peropsin in the lamprey genome.**
(DOC)
