# Supplementary figures and images for: Origin and Evolution of Retinoid Isomerization Machinery in Vertebrate Visual Cycle: Hint from Jawless Vertebrates
Source: PLoS One. 2012 Nov 27;7(11):e49975. doi: 10.1371/journal.pone.0049975 (PMC3507948; doi:10.1371/journal.pone.0049975)

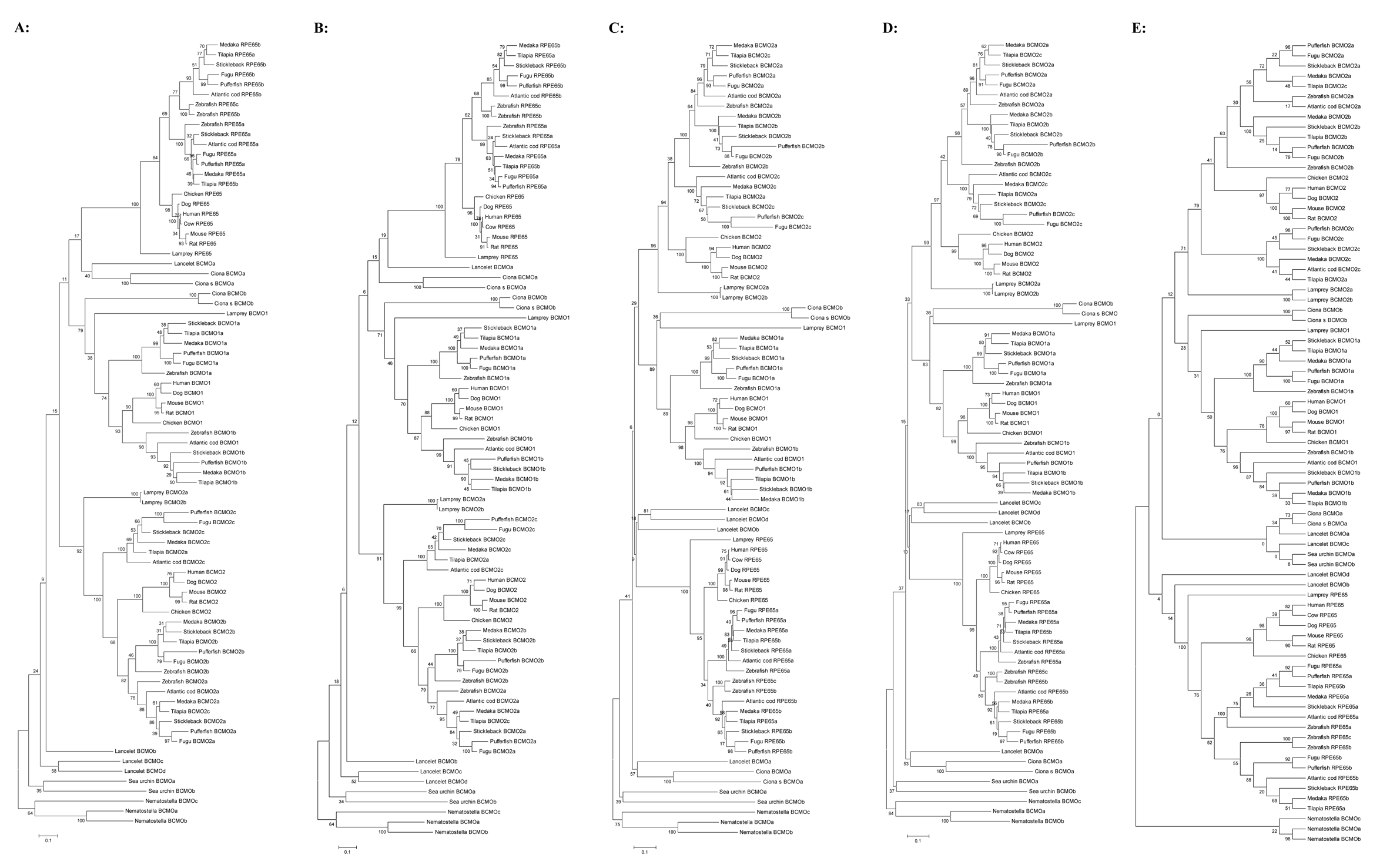

Supplement: Figure S1 — Phylogenetic trees of the BCMO/RPE65 superfamily. This shows tree topologies reconstructed using different phylogenetic methods. The numbers for the interior branches refer to the bootstrap values with 1,000 pseudoreplicates. Ciona_s stands for Ciona savignyi. A: ML, maximum likelihood phylogenetic tree, the WAG substitution model (this is the full version of Figure 1 without collapsing of the RPE65, BCMO1 and BCMO2 clades); B: ML, maximum likelihood phylogenetic tree, the JTT substitution model; C: NJ, neighbor-joining, the JTT substitution model; D: ME, minimum evolution, the JTT substitution model; E: MP, maximum parsimony. (TIF) [file pone.0049975.s001.tif]

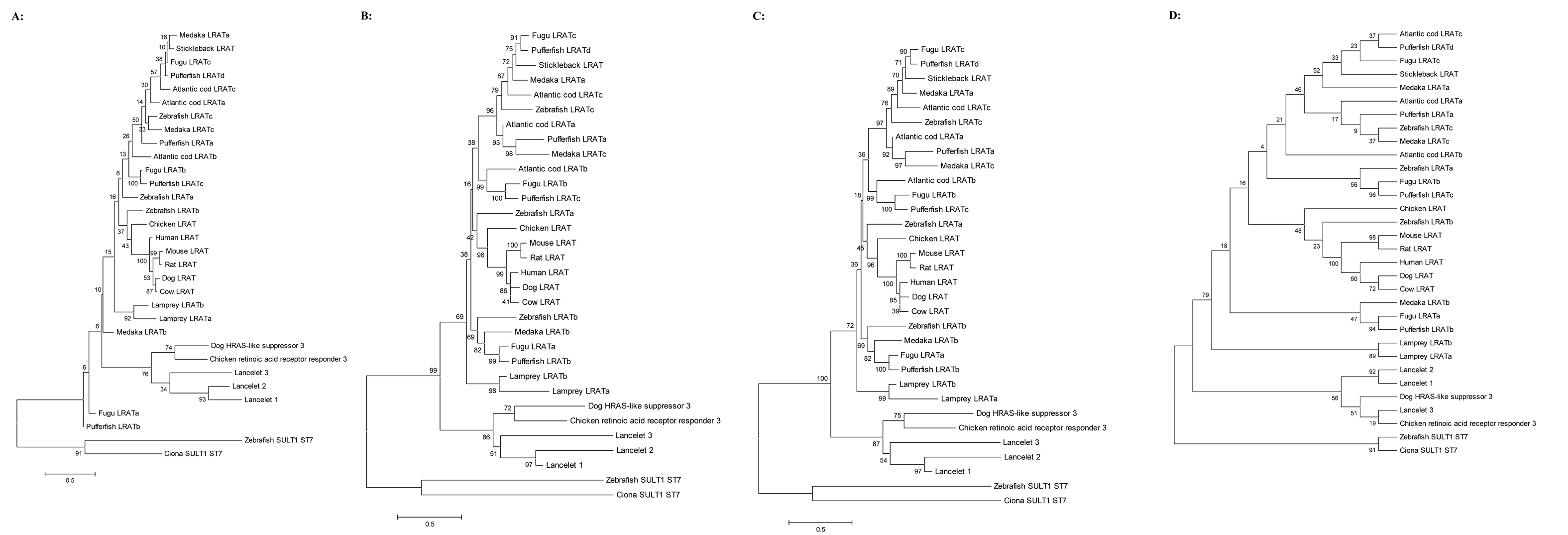

Supplement: Figure S2 — Phylogenetic trees of the LRAT superfamily. This shows tree topologies reconstructed using different phylogenetic methods. The numbers for the interior branches refer to the bootstrap values with 1,000 pseudoreplicates. A: ML, maximum likelihood phylogenetic tree, the JTT substitution model; B: ME, minimum evolution, the JTT substitution model; C: NJ, neighbor-joining, the JTT substitution model; D: MP, maximum parsimony. (TIF) [file pone.0049975.s002.tif]

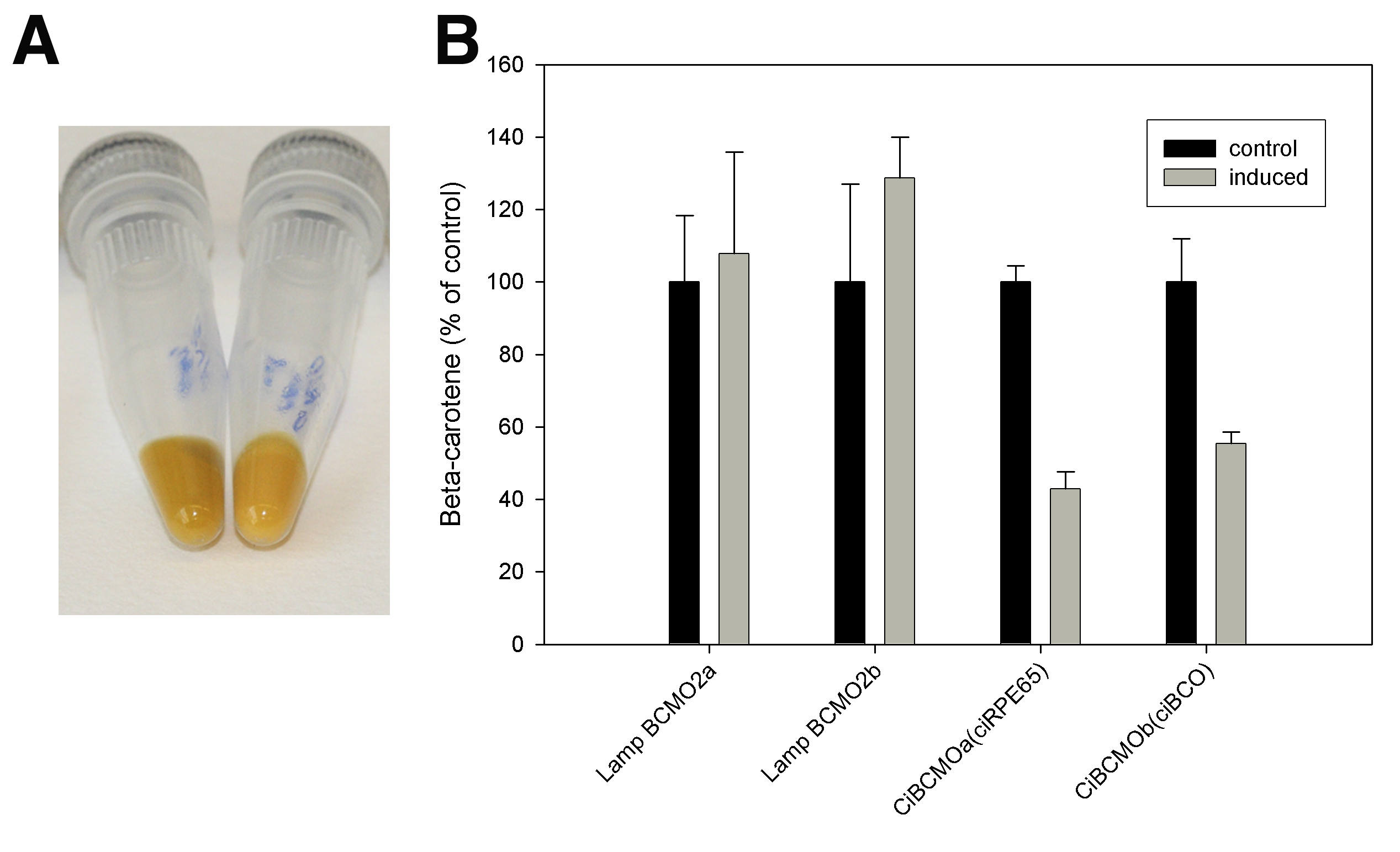

Supplement: Figure S3 — Color shift due to the cleavage of β-carotene in E. coli. A. This illustrates the color shift of the β-carotene-producing and -accumulating E. coli strain from orange to light yellow caused by the cleavage by BCMOa (Ci-RPE65) enzymatic activity of β-carotene to form apocarotenoids. While the induction of BCMOa (Ci-RPE65) expression partially bleaches the induced E. coli β-carotene strain within 18 hours (right tube), the uninduced CiRPE65 transformed culture remains orange (left tube). B. Quantification of β-carotene degradation in β-carotene-accumulating E.coli. Separate replicate cultures of cells were transformed with Lamprey BCMO2a, Lamprey BCMO2b, Ciona BCMOa (ci-RPE65), or Ciona BCMOb (ci-BCO), grown to OD600 = 0.6, split in half, then one-half was induced with 0.02% arabinose and each half allowed to grow overnight. Then cells were collected and β-carotene and its degradation products were extracted and analysed by reverse phase HPLC as described in Materials and Methods. (TIF) [file pone.0049975.s003.tif]
