# Supplementary material for: Origin and Evolution of Retinoid Isomerization Machinery in Vertebrate Visual Cycle: Hint from Jawless Vertebrates
Source: PLoS One. 2012 Nov 27;7(11):e49975. doi: 10.1371/journal.pone.0049975 (PMC3507948; doi:10.1371/journal.pone.0049975)
Supplement: Table S1 — Number of identities in triple and pairwise alignments of mouse BCMO2, BCMO1 and RPE65. T-coffee alignment of the three proteins, taking gaps into consideration. (DOC) [file pone.0049975.s004.doc]

**Table S1:**

**Number of identities in triple and pairwise alignments of mouse BCMO2, BCMO1 and RPE65.** T-coffee alignment of the three proteins, taking gaps into consideration.

| Compared alignments | Total identities | Triple/Pairwise identities |
| --- | --- | --- |
| Triple | 160 | 0 |
| RPE65/BCMO2 | 224 | 64 |
| BCMO1/BCMO2 | 224 | 64 |
| RPE65/BCMO1 | 215 | 55 |
