# Supplementary material for: Origin and Evolution of Retinoid Isomerization Machinery in Vertebrate Visual Cycle: Hint from Jawless Vertebrates
Source: PLoS One. 2012 Nov 27;7(11):e49975. doi: 10.1371/journal.pone.0049975 (PMC3507948; doi:10.1371/journal.pone.0049975)
Supplement: Table S2 — Best BLASTP hits of human RGR and peropsin in the lamprey genome. (DOC) [file pone.0049975.s005.doc]

**Table S2:**

**Best BLASTP hits of human RGR and peropsin in the lamprey genome.**

| Lamprey/Ciona/lancelet sequence, best hits of human RGR and peropsin | Vertebrate sequence | % Identity | BLASTP E-value |
| --- | --- | --- | --- |
| Control: symmetrical best hits, Ciona RGR (ci-opsin3) | | | |
| Ciona RGR NP_001027640.1 | RGR (human NP_001012738) | 26% | 10^-16^ |
| Control: symmetrical best hits, lancelet peropsin | | | |
| Lancelet peropsin XP_002605849 | Peropsin (human NP_006574) | 42% | 10^-80^ |
| Best three human RGR hits in the lamprey genome | | | |
| ENSPMAP00000004291 | Opsin 3 (human AF140242_1) | 50% | 10^-107^ |
| ENSPMAP00000007069 | Melanopsin,opsin 4 (human AAI13559) | 58% | 10^-160^ |
| ENSPMAP00000003113 | Parapinopsin (no human ortholog, frog CAG06878.1) | 53% | 10^-53^ |
| Best three human peropsin hits in the lamprey genome | | | |
| ENSPMAP00000007069 | Melanopsin, opsin 4 (human AAI13559) | 58% | 10^-160^ |
| ENSPMAP00000004291 | Opsin 3 (human AF140242_1) | 50% | 10^-107^ |
